# Tinea Capitis in a Healthy Adult: An Unexpected Diagnosis Made on Dermoscopy

**DOI:** 10.5826/dpc.1104a83

**Published:** 2021-10-01

**Authors:** Enzo Errichetti, Cinzia Buligan

**Affiliations:** 1 Dermatology Institute, “Santa Maria della Misericordia” University Hospital, Udine, Italy

## Case Presentation

**Figure 1:**
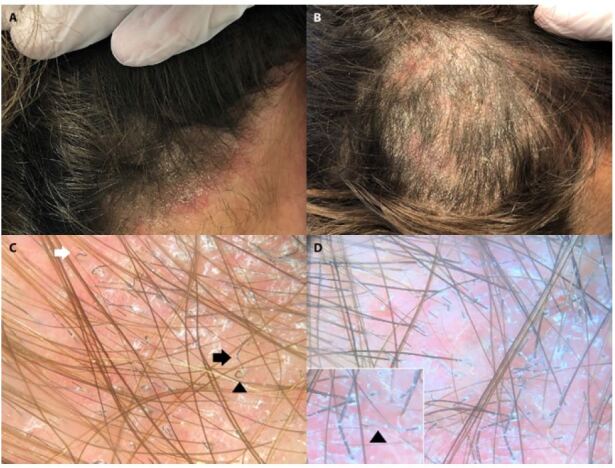
(A, B) Clinical examination shows erytheamatous-desquamative patches of the scalp. (B) Broken hairs are also visible. (C) Dermoscopy (Polarized light; ×10 magnification) reveals unspecific erythema and white scales along with *comma* hair (black arrowhead), *corkscrew* hair (white arrow) and *zigzag* hair (black arrow). (D) *Morse code* hair (hair shafts presenting alternating whitish and brown bands) (black arrowhead in the inset) are also present.

A 37-year-old healthy male presented with a 2-month history of itchy, scaly, erythematous patches on the scalp, displaying broken hairs in some areas (Figure 1, A and B). Medical history (including immune system/infectious diseases) was unremarkable, and no medication was being taken. At a first glance, the main clinical suspect was scalp psoriasis with broken hairs resulting from continuous scratching. Besides broken hairs, erythema and white scaling, dermoscopic examination revealed findings contrasting with the clinical suspect, ie *comma* hair, *zig-zag* hairs, *corkscrew* hair and *Morse code* hair (Figure 1, C and D). A fungal culture from lesional scales was performed and *Microsporum Canis* was isolated, consistently with the diagnosis of tinea capitis. An 8-week course of oral Terbinafine 250 mg/die was prescribed with complete healing.

## Teaching Point

Tinea capitis is typically considered a disease of childhood or immunosuppressed patients, and postmenopausal women, yet healthy adults may also develop it [1]. The rarity of the condition in this category of patients is often responsible for diagnostic delays or mistreatments [1]. In this regard, dermoscopy may increase the index of suspicion by showing specific features resulting from fungal invasion of the hair shaft which may cause a structural weakness of the infected hairs, with consequent more or less marked bends (viz. *comma*, *corkscrew*, or *zig-zag* hair) or irregularly narrowed pale areas (*Morse code* hair) [2].
